# On Dysentery

**Published:** 1831-04-01

**Authors:** 


### ON LARGE DOSES OF IPECACUANHA IN DYSENTERY. By *W. Twining*, Esq.

[Calcutta Med. and Phys. Trans., Vol. IV.]

A CONSIDERABLE portion of the last volume of the Calcutta Transactions is taken up with a very detailed report on dysentery by Mr. Twining and Dr. Mortimer. This is a disease not very common in this country, though a severe scourge of tropical climates—and even of many parts of Europe. We shall therefore give a brief account of the mode of practice which Mr. Twining has employed with success in the above disease. He premises that he does not mean to hold up any one remedy as applicable to all stages of dysentery; but freely admits the general principles on which venesection and other means are employed in the early or inflammatory stages of the disease. Consistent with these principles, however, he can strongly recommend "very large doses of ipecacuan" for the purpose of relieving the tenesmus and irritation, restoring a healthy state of the intestinal secretions, and promoting the healing of ulcerations when they exist.

"To fulfil these intentions, the powdered ipecacuanha has been exhibited combined with a bitter extract, whereby vomiting is counteracted. I have generally used the extract of gentian for this purpose, and by prescribing these two remedies in nearly equal proportions, we may, if requisite, give from six to twelve grains of ipecacuanha without often exciting nausea.

The quantity in which many medicines are taken, determines not only the intensity or quantum of effect, but the mode of action; and the organs and functions on which those actions are directed. This seems particularly the case with ipecacuanha, and the observations at present made induce me to believe, that the most effectual dose of this medicine, when intended to act as an anti-dysenteric, is six grains for an adult: larger doses sometimes produce general uneasiness, and have not been often employed by me in dysentery." 171.

In acute dysentery, the first object (after adequate bleeding) is to clear the bowels by a dose of castor oil, and then to administer six grains of ipecacuanha with four grains of gentian and five grains of the pilula hydrargyri every night at bed-time, and also at day-light in the morning; while one drachm of the compound powder of jalap (℈j. of jalap to ℈ij. cream of tartar) is to be given about eleven o'clock in the forenoon. If the patient be of an irritable habit, half a drachm of the powder will be sufficient.

"By these means we may soon procure a cessation of all the distressing symptoms, and it is then sufficient to continue six grains of ipecacuanha with four grains of extract of gentian every night, giving a smaller dose of compound powder of jalap, or a moderate dose of castor oil every morning, for four or five days more. The above treatment generally produces a restoration of a natural state of the alvine evacuations so quickly, that patients are with difficulty restrained from using an improper quantity of food in the early stage of convalescence, when, although the evacuations from the bowels may be in a regular and natural state, the intestines must remain weak, ready to be irritated by slight causes, and incapable of digesting any thing, but the most bland food. During the treatment now detailed, great attention is requisite to the quantity of food used, as well as its quality. Not more than six ounces of food and drink together should be taken at one time, nor should this quantity be repeated oftener than is absolutely requisite to prevent the patient from sinking; in severe acute cases, half this quantity should not be exceeded. By thus strictly attending to the quantity of food and drink used, it is probable, that the ingesta will be almost entirely absorbed in the upper portions of the small intestines; so that the colon, which is the seat of dysentery, may be as nearly as possible empty while in an irritable state, and during the subsidence of disease, or the healing of ulcers. Bread and tea, or milk and sago are the most proper, to which soups in limited quantity may be added during the progress of recovery: solid food of any sort except bread not being allowed, until convalescence is far advanced. Should thirst be urgent, the patient may drink ozs. ij. of cold camomile tea, but ought not to repeat this oftener than once in an hour, however urgently required; it is the best drink that can be used, and does not increase thirst.

When this plan of medicine and diet is followed, the ipecacuanha seldom produces vomiting; but a healthy state of the bowels is speedily restored. In a few cases, six grains of ipecacuanha combined with gentian have caused vomiting, but this circumstance has been exceedingly rare; and it has often happened, that patients were not sensible they had taken medicine possessing nauseating qualities. In more than one instance, medical men were subject to the same treatment for dysentery, and expected that half the quantity of medicine taken would make them vomit; still they experienced no such effect: being equally surprised at the exemption from vomiting, and the early and complete relief from suffering and dysenteric symptoms which the remedy effected. Twelve grains of ipecacuanha have been often given to adults combined with eight grains of extract of gentian, in four pills, without exciting vomiting; and half that quantity has been taken by a child thirteen years of age without causing nausea. I have never known five grains of ipecacuanha combined with the same quantity of gentian in pills, nauseate an adult. Some persons who were watching the effects of these large doses, expressed doubts as to the quality of the ipecacuanha, in use at the General Hospital; therefore, for the purpose of removing any chance of fallacy on this point, three grains of the medicine from the same bottle were given without the gentian, and it produced free vomiting, this was repeated several times so as entirely to satisfy those who attended to the experiment. Some other combinations of the same remedy produce a similar effect, appearing to divert the action of ipecacuanha from the stomach and to direct it towards other organs; as is well known to be the case when a moderate dose of jalap is given with as much ipecacuanha as would alone excite vomiting, instead of which the combination acts as a mild and satisfactory purge; this effect is rendered more certain if a small quantity of cream of tartar or other vegetable acid be added. A consideration of these established facts, might induce us to try other combinations for the same purpose, when gentian is not procurable. It is of importance that the extract of gentian be not burnt, or otherwise impaired in quality, for then it will not have the desired effect in preventing vomiting." 177.

In many of the slighter cases of dysentery, ipecacuan and gentian may be given night and morning, without the blue pill, a small dose of the jalap powder being exhibited daily at noon. "But it has appeared to me that the efficacy of the other remedies is very much increased by the blue pill."

The first effects of this plan, in ordinary cases of acute dysentery, are generally a slight increase of the secretion from the bowels, the evacuations becoming more copious and fæculent—with an abatement of the pain and tenesmus—and a diminution in quantity of the blood and slime. Mr. T. has often noticed that a change takes place in the specific gravity of the fæces voided—a sediment appearing at the bottom of more fluid stools. This sediment is frequently a light gray powder resembling bran. Occasionally it is more dense, like pounded slate—more rarely like split peas. These appearances almost invariably indicated that a favourable change was at hand.

When the above treatment was steadily pursued for a few days, scarcely any other remedies were requisite, when the patients applied at an early stage of the disease. At the same time Mr. T. properly recommends bleeding by leeches as well as the lancet, in all cases of recent dysentery, where there is pyrexia, morbid sensibility of the abdomen on pressure, evacuation of blood by stool, or tenesmus. When depletion has done all that can be expected from it, then the plan above-mentioned " will be found of infinite service in soothing irritability, and restoring a healthy state of the bowels." Tepid baths, fomentations, &c. are proper auxiliaries. Mr. T. thinks that opium is generally injurious in this disease, unless when combined with calomel, so as to cause that medicine to be retained in the first portion of the intestines, and thus act on the hepatic and duodenal secretions.

Our author having only employed this plan in the lower provinces of Bengal, he can only speak of it in that climate. A great number of cases are adduced; but these we shall pass over. They corroborate the author's statements, as a matter of course, else they would not be brought forward. We shall extract the following note respecting hepatic dysentery.

"The frequency of *Hepatic Dysentery* in tropical climates is very generally admitted. In my observations for the purpose of ascertaining the relation of hepatic disease and dysentery, and the periods at which the one follows the other; I have in many cases noticed symptoms which induced me to believe, that hepatic irritation and congestion may be caused by extensive disease of the great intestine. In these instances there had been no evident symptoms of liver disease, until the dysentery had existed many days: in some cases the bowel complaint was on the decline, and the quantity of evacuations considerably decreased, when attention was called to the state of the liver, by the occurrence of some tumefaction at the right hypochondre, and a tenderness on pressing over the liver; the patient has complained of a sense of tremor and uneasiness in the belly, which has been attended by slight pyrexia, general restlessness, a hectic flush in the cheek, and some irritability of mind: the evacuations being then generally scanty in quantity and of deep orange colour inclining to red; more rarely pain in the top of the right shoulder has supervened. For this state a few leeches have been applied, and repeated with benefit, although the patients were weak; but most commonly these symptoms have subsided in three or four days, on giving daily some doses of calomel and rhubarb in the morning and dr. i. of sulphur in the afternoons, restricting the diet to tea, bread, and sago, *in very small quantities*. The calomel and rhubarb seem to solicit an increase of the biliary secretion, whereby the congestion is relieved, while sulphur given in a small quantity of water is a mild aperient, affecting chiefly the colon; a considerable part of this medicine is voided almost unchanged, and it has appeared to me to act favourably as a local application to the ulcerations of the great intestines. It is perhaps hardly safe to advance opinions concerning the state of internal organs in disease, without the support of repeated dissections. But such symptoms, as above described, have occurred so frequently, and in the order now related, that I cannot help ascribing them to hepatic irritation and congestion; the former arising from a disordered state of the blood which is returned by the veins of the diseased intestine to circulate through the liver, the latter dependant on suppression of the purging. These sentiments regarding hepatic disease, as occasionally excited by ulcerated colon, require the confirmation of much more numerous observations; but at present they seem sufficiently established to claim attention." 200.

The paper concludes with a communication from Dr. Mortimer, of the Madras General Hospital, on the use of ipecacuan combined with calomel in dysentery, as seen in stations where a very hot atmosphere prevails, and the soil is dry and sandy. The following extract contains the pith and marrow of Dr. Mortimer's letter.

"The first remedy is bloodletting, general and topical, or either, the extent being determined by the effects produced. The former is considered as more particularly indicated, when the state of the pulse manifests constitutional excitement; the patient being always kept in an erect position during the operation; while the latter is had recourse to, with reference to the urgency of local symptoms; a dose of castor oil is then administered, and the appearance of the alvine discharge is the symptom by which the extent to which it may be necessary to exhibit mercury is determined. In some cases, especially where the patient had been admitted at night, and his motions not been seen, a dose of calomel, to the extent of ten grains or perhaps more, has been given before the oil, in anticipation of structural derangement of the liver, in general, existing to a greater or less degree in dysentery. The state of the hepatic discharge then, as indicated by the appearance of the alvine evacuations, regulates the exhibition of mercury, and the preparation preferred, in the course of the treatment, is blue pill. At the commencement of treatment, the quantity usually prescribed is five grains three times a day, and it has been seldom or ever necessary to continue it to the extent of producing ptyalism,—a uniform discharge of healthy bile being the object to be attained. The consideration of the comparatively small quantity, by which this has been in general produced, when at the same time the ipecacuanha was administered in the manner about to be adverted to, may, perhaps, fairly admit of the opinion of a favourable influence being exerted by the latter, in promoting the effect of the mercury, and rendering a less quantity necessary for correcting vitiated secretion of the liver, and restoring a flow of healthy bile."

"The use of the ipecacuanha is generally commenced after the bleeding, or five or six hours after admission, and given in doses of five grains each, in combination with an equal quantity of powder of gum acacia, every hour or second hour, as the patient's stomach may be able to bear it without producing vomiting. During its exhibition, the use of fluids is limited as much as possible, and the patient generally complains of a good deal of nausea, although not so much as might be anticipated, and its rejection by the stomach is by no means a common occurrence.

The immediate effect of the acacia, or why vomiting is not so readily produced, when the ipecacuanha is given in combination with it, as when exhibited alone, I do not pretend to explain; being satisfied by the experience of having exhibited a much larger quantity of it, in the manner alluded to, than I have been able to administer by itself, without producing the effect in question. In some cases it has been given to the extent of between 90 to 100 grains in 24 hours." 213.

The above paper is a valuable, because a practical one, and may prove beneficial in many other countries than the peninsula of India, or the marshy plains of Bengal.

